# On the Transmission of a Predisposition to Diseases by Inheritance

**Published:** 1871-09

**Authors:** T. B. Smith


### ARTICLE VII.

### *On the Transmission of a Predisposition to Diseases by Inheritance.*

By T. B. Smith, M. D.

The transmission by inheritance of a predisposition to disease, is the result of the same general law of nature which gives form and features of progenitors to their offspring. There is an ingrafted tendency in all living organized matter to reproduce itself. "Like begets like." From this it must follow as the relation between cause and effect that the subjects of unsound organism, and feeble vital forces cannot propagate healthy decendants. The same thing occurs, but less obviously to common observation, in the the mental and moral world. An unsound condition either of body or of mind is very likely, nay, almost certain, to be perpetuated in the offspring of such parents. This law may be very much modified by various counteracting influences.

For example, there may be in parentage two opposing forces or causes, bane and antidote. Thus, the paternal side may be of unsound organism and feeble constitution, with actual hereditary disease in his system, while upon the maternal side there may be firm health and a highly vitalized system, with no hereditary taint in her organization. The offspring of such a parentage would probably escape the disease of the father, the healthy tendencies imparted by the mother rendering inoperative, diluting or attenuating the *causi morbi* transmitted by the father, though it might possibly crop out sooner or later, in some one of his descendants, particularly if the element thus transmitted were tuberculous.

The hereditary diathesis may undoubtedly be transmitted from parent to child, and yet remain in a quiescent state perhaps for several generations, the circumstances and conditions which would tend to develope it in to active disease not occurring; and diseases regarded as hereditary may be acquired in the absence of any predisposition to them, the conditions which develop an hereditary disease originating it; and when originated a momentum is acquired which is transmissable by inheritance.

First in importance in the list of hereditary diseases, and the one least under the control of remedial measures, is pulmonary tuberculosis. It prevails in all parts of the globe, and in all climates. It is said to carry off one fourth of the inhabitants of Europe, and that estimate would probably not be too much for this country. It is of high antiquity, and has prevailed with almost equal fatality in all ages of the world.

When the tubercular diathesis is manifested in the form of enlarged and indurated tonsils, and swelling and suppuration of the cervical gland, it is thought to avert its development in the lungs, persons thus affected in childhood seldom dying of pulmonary consumption, the morbid material upon which the disease depends seeming to be exhausted or eliminated from the system in that way.

Though the hereditary diathesis in phthisis pulmonalis is more marked and its fatal results more certain than in most of the transmitted predispositions to disease, there are other inherited tendencies equally dreaded, the cancerous diathesis much more, and the constitutional tendency to insanity and intemperance quite as much.

These diseases, as before remarked, are no doubt often the result of accidental causes: hardship, exposure and imperfect nutrition may induce consumption in a sound constitution; a blow upon the female breast may originate cancer of that organ; insanity may be the result entirely of moral causes, and intemperance induced solely from the acquired habitual use of intoxicating drinks, without any particular constitutional tendency thereto. But that these maladies are all transmissable by inheritance observation has established beyond a doubt, and in a great majority of cases they are the direct result of an hereditary diathesis. Habitual intemperance has come to be regarded as an actual constitutional disease, known as chronic alcoholism.

The constant action upon the animal economy of so deleterious an agent as alcohol in large quantities cannot but produce very great and serious organic changes, which it is well known it does produce in the stomach, the liver, brain and kidneys, and in fact changing the whole nature, physical and moral. That such a condition in a parent should impart to the offspring a predisposition to like results is in accordance with the general law of transmitted tendencies.

The hereditary diathesis can never be eradicated from the system; it is engrafted upon the very nature. Hereditary disease may be arrested or retarded, but the predisposition to it will ever remain. This class of diseases are most of them but very little under the influence of remedial agents; there are no known specific remedies for the cure of consumption. Medication has no doubt been productive of more harm than good in the treatment of this disease. Hygienic measures seem to exert more influence over it than medicine Out door exercise, a generous diet and the various other hygienic means tending to promote health are more to be relied upon than any other remedial measures. It is doubtful indeed whether a change of climate exerts any beneficial influence except in so far as it conduces to a change of habits, as regards exercise and outdoor life.

In the treatment of cancer it is said that a specific has been discovered in a plant growing in the mountains of Ecuador, in South America, the cundurango. It has been used, it is said, with success in this country, in the treatment of many cases of this disease. But I judge it has not yet had a sufficient trial to establish its claim as a specific in this malignant malady.

It is believed by some enthusiastic minds that among nature's hidden resources there will yet be discovered a "panacea for all the *ails* which flesh is heir to." But this Utopian dream will probably never be realized; good and evil, health and disease have ever been coexistent, and unless the order of nature is changed, must ever continue to be.

Insanity and intemperance as such seldom come under treatment in private practice. These cases are generally committed to public institutions for treatment—the insane and inebriate asylums—from their adaptation to the employment of the improved means which an advanced science and an enlightened humanity have suggested, in the treatment of these cases.—*Med. and Surg. Reporter.*

---

